# Molecular Dynamics Study on the Adsorption and Modification Mechanism of Polymeric Sand-Fixing Agent

**DOI:** 10.3390/polym14163365

**Published:** 2022-08-18

**Authors:** Wei Huang, Xueyu Geng, Jing Li, Cuiying Zhou, Zhen Liu

**Affiliations:** 1 Guangdong Engineering Research Centre for Major Infrastructure Safety, School of Civil Engineering, Sun Yat-sen University, Guangzhou 510275, China; 2 School of Engineering, University of Warwick, Coventry CV4 7AL, UK; 3 Key Laboratory for Polymeric Composite and Functional Materials of Ministry of Education, School of Chemistry, Sun Yat-sen University, Guangzhou 510275, China

**Keywords:** molecular dynamics, polymeric sand-fixing agent, adsorption mechanism, sandy soil improvement, microscopic mechanism

## Abstract

Chemical sand-fixing technology has shown good potential in preventing desertification, but the effect is determined by materials. In this paper, the adsorption behavior of quartz and six common polymer sand-fixing agents under dry conditions was studied by molecular dynamics method. The results show that all polymers could be adsorbed on the surface of quartz and their functional groups play an important role in the adsorption process. Compared with other materials, the binding energy and the number of hydrogen bonds of PAA-quartz composites were improved by 30.7–65.6% and 8.3–333.3%, respectively. It was found that the number of hydrogen bonds formed under the unit molecular was positively correlated with the mechanical properties of the improved sandy soil. This study provides an accurate, efficient and inexpensive qualitative evaluation method for the curing effect of sand fixers, which will assist in the screening and development of new high performance sand fixers.

## 1. Introduction

Frequent human activities have led to ecological damage and the expansion of desertification. Land desertification has become an important environmental problem restricting the development of human society [1]. Therefore, some measures are needed to protect the sand surface in order to control or delay land desertification. Among current technologies, chemical sand-fixation technology has been widely used because of its effectiveness, quick effect and simple operation [2]. As a kind of chemical sand-fixing agent, polymer sand-fixing agents, as a potential alternative of chemical sand-fixers, are widely used in desertification control due to its good adhesion and film-forming ability [3,4,5]. However, the performance of different types of polymeric sand fixers varies significantly. To achieve better sand fixation performance in practical applications, the dosage of different types of sand fixers also varies greatly. This means it is difficult to evaluate their effectiveness under the same criteria, which further makes it challenging to screen and develop new high-performance sand fixing agents.

Molecular dynamics simulation as a new scientific research method can quantify the interaction between different materials, so as to help people better understand the interaction mechanism between different materials [6,7,8]. Quezada et al. [9] analyzed the adsorption behavior of HPAM on the quartz surface by molecular dynamics simulation, and found that the increase in salinity was conducive to the formation of collisions between the initial aggregates, but it also led to the decrease in the rotation radius of the polyelectrolyte, resulting in the limitation of its bridging ability. Anastassiou and Mavrantzas [10] compared the adsorption behavior of two model pressure-sensitive adhesive (PSA) materials on the surface of the three materials. It was found that the binding ability of the polymer on the surface of α-quartz and α-Fe_2_O_3_ was significantly stronger than that of α-Fe. It shows that the introduction of oxygen atoms into the metal surface or polymer molecules will significantly increase the adsorption energy. Duan et al. [11] studied the adsorption mechanism of N-dodecylethylenediamine (ND) on quartz surface, especially the role of functional groups (-NH-) in the adsorption process. The results show that the protonated group with positive charge can be adsorbed on the quartz surface by electrostatic attraction, and the hydrogen of the protonated polar group can form hydrogen bonds with the oxygen on the quartz surface. Lan et al. [12] studied the adsorption of violanthrone-79 (VO-79) in different organic solvents (n-heptane, toluene, and heptol with three different n-heptane/toluene volume ratios) as a model asphaltene compound on the quartz surface. The results showed that the solvent type had a great influence on the adsorption rate and final adsorption amount. High energy analysis shows that the adsorption of polymer on quartz surface is mainly driven by van der Waals force, accompanied by electrostatic interaction, hydrogen bond and solvation free energy. It can be seen from the above studies that the research on polymer/quartz adsorption mechanism based on computer simulation has made gratifying progress, and many important conclusions have been obtained [13]. However, in the field of sand solidification, the mechanism of molecular scale research works still remains in its infancy and the analysis combined with mechanical test to date has not been reported. In view of the importance of interaction between sand-fixing agent and sand particles, it is of great theoretical significance to study the adsorption effect and mechanism of sand-fixing agent by molecular dynamics method. It is of great engineering significance to combine the simulation results with the mechanical experimental data to realize the qualitative evaluation of the sand fixation effect.

In the field of sand consolidation, the lack of understanding of the mechanism at the molecular level is detrimental to the development of new sand fixing agents. In this paper, molecular dynamics software was used to study the adsorption behavior of quartz and six common polymer sand-fixation agents (polyvinyl acetate (PVAc), polyurethane (PU), polyaspartic acid (PASP), hydroxypropyl methyl cellulose (HPMC), polyacrylic acid (PAA) and polyacrylamide (PAM)) under drying conditions. The objective of this study is to understand and predict the microscopic structure characteristics (i.e., density distribution and hydrogen bond number) and macroscopic thermodynamic properties (i.e., adsorption work) of polymers on the surface of substrates through computer simulations, thus achieving the goal of rapidly evaluating the effectiveness of different types of polymers on sandy. This study provides an accurate, efficient and inexpensive qualitative evaluation method for the curing effect of sand fixers, which is helpful for the screening and development of new high performance sand fixers.

## 2. Research Contents and Methods

### 2.1. Material Selection

According to our team’s previous experiments and references, it is known that functional groups play a decisive role in the properties of organic compounds [14,15,16,17]. Therefore, through literature review, the following six polymers were selected for simulation based on the functional group types in the widely used polymer sand-fixation agents. The detailed information of selected polymers is shown in Table 1. They possess the following functional groups alone or simultaneously: ester group, carbonyl group, peptide bond, hydroxyl group or amide group. The simulation results of different types of functional groups in the adsorption process on polymers can help us to understand the role and possible impacts.

### 2.2. Molecule Models

Molecular dynamics simulation is a calculation method for numerical analysis using computer. Various physical and chemical properties of molecular system are obtained by establishing molecular models at atomic level, simulating molecular structures and analyzing behaviors under certain force field conditions [25]. COMPASS (condensed-phase optimized molecular potential for atomistic simulation studies) force field is a force field with good universality. It is the first force field based on ab initio calculation and empirical data parameterization. The structure, conformation, vibration and thermodynamic properties of isolated and condensed molecules can be accurately predicted. Experiments show that this method can be applied to the study of organic and inorganic systems [26,27]. In this paper, COMPASS force field was used to describe the oligomers of PVAc, PU, HPMC, PASP, PAA, PAM and SiO_2_ crystals. Each oligomer consists of 60 carbon (C) atoms and several other atoms to ensure that each oligomer has a close molecular weight. Their atomic types and definitions in force field are shown in Table 2. The initial structure is optimized to minimize its energy. Solid substrate was simulated by quartz (0 0 1) crystal plane. All non-bridging oxygen atoms were protonated in order to obtain a chemically real surface.

In practical engineering, the dosage of sand-fixing agent is often calculated by relative mass (polymer mass/sand mass). Therefore, the use of close molecular weight to simulate polymer adsorption can be better compared with the macro-scale improvement effect. In this paper, when building the periodic unit cell model of oligomers, we first ensure that the number of carbon atoms in the main chain is the same, and more than part of the carbon atoms are manually removed and H atoms are added to maintain the overall electrical balance of oligomers.

### 2.3. Simulation Details

The periodic cell models of oligomers PVAc, PU, HPMC, PASP, PAA and PAM were built by using the amorphous cell module in Materials Studio. The energy of the initial structure is high, and geometry optimization in forcite module is selected to obtain the low energy form of the structure. The initial velocity of atoms in the system is randomly selected by Maxwell–Boltzmann distribution at initial temperature. In order to avoid the influence of the boundary on the adsorption process, the periodic boundary conditions were used in the simulation. The NVT ensemble was used, the temperature was set at 298 K, and the Nose–Hoover method was used to control the temperature. The simulation time is 20 ns. When the total energy of the system converges, it is judged that the system reaches the equilibrium state. On this basis, the relative density distribution, hydrogen bond and binding energy were calculated. To avoid trapping in a single potential well, the polymer is placed at least 5 Å above the quartz surface at the beginning of the simulation. At this position, each polymer is rotated in 10 different directions, and each axis is rotated by 90°. The binding energies and hydrogen bonds of all configurations were analyzed. The maximum hydrogen-receptor distance and the minimum donor-hydrogen-acceptor angle were the default value of the software (2.5 Å and 90°).

## 3. Results

### 3.1. Relative Density Distribution of Atoms on Quartz Surface

**Figure 1:**
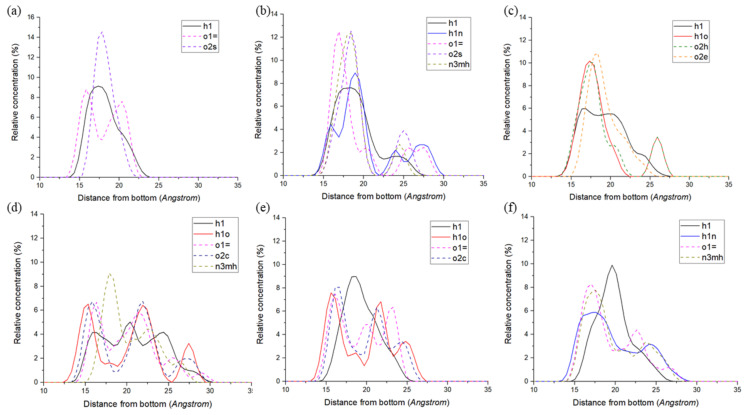
Density distribution of six polymers in the direction perpendicular to the surface of quartz crystal (z direction): (**a**) PVAc, (**b**) PU, (**c**) HPMC, (**d)** PASP, (**e**) PAA, (**f**) PAM.

In order to analyse the polymer structure near the quartz surface, the relative density distributions of different atomic species of six polymers perpendicular to the surface are calculated, as shown in Figure 1. Defines the surface position as a surface perpendicular to the xy surface and z = 0. By analyzing the relative density profile of atoms in the z direction, it can be found that the adsorption of polymer molecules on the surface has a certain degree of similarity. Among the six polymers, the first density peak of o1= in PVAc (Figure 1a) is located at the position closest to the surface, indicating that the ester group plays an important role in the adsorption process. In PU (Figure 1b), the first density peaks closest to the surface were h1n, n3mh and o1=, which were provided by peptide bonds; The first density peaks of h1o and o2h in HPMC (Figure 1c) were closest to the surface, indicating that hydroxyl in HPMC played a major adsorption role. In PASP (Figure 1d), the closest order of the first density peak to the surface was h1o, o2c, h1 and o1=, combined with the molecular formula of PASP, it can be found that the elements are provided by peptide bonds and carboxyl groups, respectively. h1o and o1= of the first density peak closest to the surface in PAA (Figure 1e) were both from carboxyl. In PAM (Figure 1f), the elements closest to the surface of the first density peak are h1n, o1= and n3mh, indicating that the amide group dominates the adsorption process of PAM.

### 3.2. Number of Hydrogen Bonds Formed by Polymer on Quartz Surface

**Figure 2:**
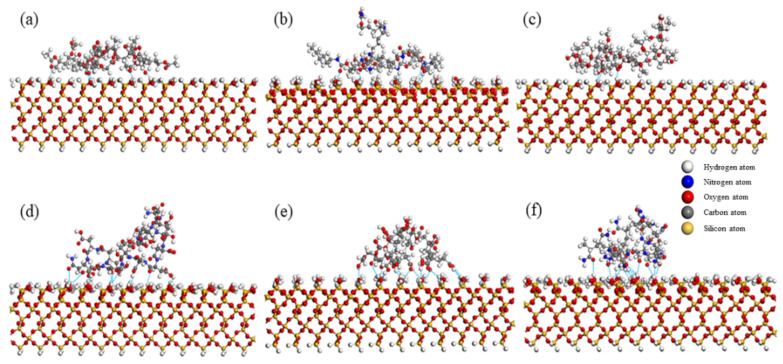
Pictures of polymer molecules adsorbed on a quartz surface, with hydrogen bonds shown in dashed lines. (**a**) PVAc, (**b**) PU, (**c**) HPMC, (**d**) PASP, (**e**) PAA, (**f**) PAM.

Hydrogen bond, as a special intermolecular force, also plays an important role in the surface adsorption process. The adsorption configurations of PVAc, PU, HPMC, PASP, PAA and PAM with different configurations on the quartz surface under vacuum conditions were analyzed by using the hydrogen bond analysis module of MS, and the number of hydrogen bonds were calculated (Figure 2). The results showed that the number of hydrogen bonds in PVAc, PU, HPMC, PASP, PAA and PAM systems with the lowest energy was 5, 5, 3, 10, 13 and 12. Hydrogen bonds are mainly formed by ester, carbonyl, peptide, hydroxyl or amide groups in polymer molecules, which is consistent with the density profile analysis (Figure 1). This result show that functional groups play an important role in the interaction between polymer molecules and quartz surface.

### 3.3. Binding Energy of Polymer on Quartz Surface

**Figure 3:**
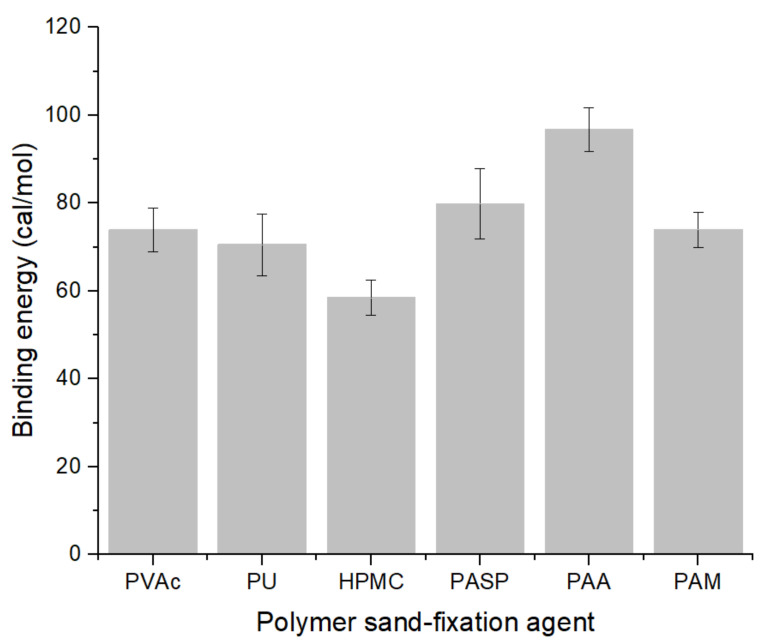
Binding energy of polymer sand-fixing agents.

Under vacuum conditions, the binding energy of the material is calculated according to the following formula [25]:

(1) $$E_{ads}=E_{total}-E_{polymer}-E_{quartz}$$

where $E_{total}$ is the configuration energy of the whole system containing all atoms, $E_{polymer}$ is the configuration energy of polymer molecules, $E_{quartz}$ is the configuration energy of quartz. $E_{ads}$ is the binding energy related to the interaction of polymer quartz, which determines the energy needed to break the interaction between polymer and quartz. At the same time, this value also characterizes the adhesion strength of the interface (Figure 3) [28,29]. In our definition, the more negative is the binding energy, the stronger is the interaction between oligomers and substrate. The binding energies of PVAc, PU, HPMC, PASP, PAA and PAM are −73.96 cal/mol, −70.56 cal/mol, −58.43 cal/mol, −79.86 cal/mol, −96.74 cal/mol and −74.01 cal/mol, respectively.

In summary, functional groups have a significant effect on the formation of hydrogen bonds and binding energy of polymers during adsorption on the substrate surface. The degree of influence depends on the type of functional group.

## 4. Discussion

### 4.1. Effect of Functional Groups on Hydrogen Bonds

**Figure 4:**
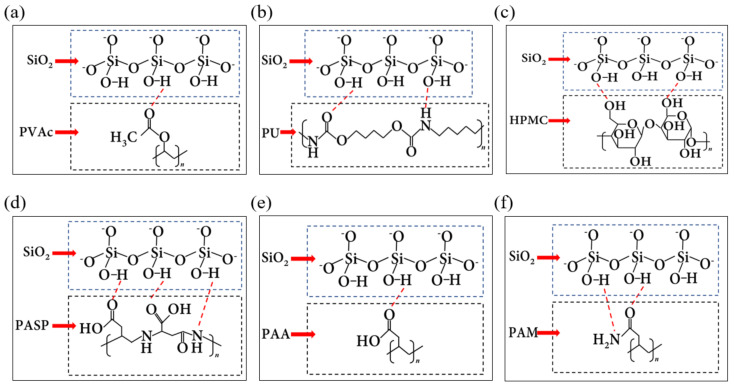
Hydrogen bonds formed by polymers (dotted lines in red in the image is the hydrogen bond). (**a**) PVAc, (**b**) PU, (**c**) HPMC, (**d**) PASP, (**e**) PAA, (**f**) PAM.

By comparing the adsorption behavior of PVAc, PU, HPMC, PASP, PAA and PAM oligomers on quartz surface, it can be seen that hydrogen bonds play a key role, but different functional groups have different stability when forming hydrogen bonds. In oligomers with only ester groups (i.e., PVAc), ester groups (−COO−) play a major role in the formation of hydrogen bonds, contributing > 99% of hydrogen bonds (Figure 4a); in oligomers with only peptide bonds (i.e., PU), peptide bonds (−NH−CO−) play a major role, contributing more than 99% hydrogen bonds (Figure 4b); in the oligomers with carboxyl groups (i.e., PAA), (−C=O) and (−OH) in the carboxyl groups can contribute 61.5% and 38.5% of the hydrogen bonds, respectively (Figure 4e). In oligomers with only hydroxyl (i.e., HPMC), hydroxyl contributed > 99% hydrogen bonds (Figure 4c); In the oligomers with amide groups (i.e., PAM), (−NH−) and (−C=O) contribute 50% hydrogen bonds, respectively (Figure 4f).

For oligomers containing both peptide bonds and carboxyl groups (i.e., PASP), peptide bonds and carboxyl groups contribute 30% and 70% hydrogen bonds, respectively (Figure 4d). The number of hydrogen bonds formed by PASP is larger than that of PU containing only peptide bonds and smaller than that of PAA containing only carboxyl groups, indicating that carboxyl groups have stronger electrostatic force between electrons than peptide bonds, thus forming hydrogen bonds more easily.

Comparing PAA and PAM with similar chemical structures, it can be found that the number of hydrogen bonds and adsorption energy of PAA are higher than those of PAM, indicating that the carboxyl group has stronger electronegativity than the amide group, and it is easier to form hydrogen bonds under the same conditions.

### 4.2. Effect of Functional Groups on Binding Energy

In the process of adsorption between a polymer and quartz surface, the more the number of hydrogen bonds formed, the lower the binding energy and the better the adsorption. However, due to the different types of atoms or functional groups, the adsorption mechanism of different polymers on quartz surface is different, resulting in different binding energies.

According to the molecular dynamics simulation of the above six materials, the binding energy of ester group on the quartz surface was slightly higher than that of peptide bond (PVAc was −73.96 cal/mol while PU was −70.56 cal/mol) when the molecular weight was close. The carboxyl group has the highest binding energy. In the case of all carboxyl groups, increasing the peptide bond will affect the polarity of the carboxyl group, resulting in lower binding energy (PASP was −79.86 cal/mol while PAA was −96.74 cal/mol). In the case of peptide bond, increasing carboxyl group will increase binding energy slightly; Although hydroxyl can also form hydrogen bonds (HPMC was −58.43 cal/mol), its binding energy is lower than that of ester group (PVAc), peptide bond (PU), carboxyl group (PAA) and amide group (PAM); Under the premise of chain structure, the binding energy of carboxyl group was higher than that of amide group (PAA was −96.74 cal/mol while PAM was −74.01 cal/mol).

### 4.3. Number of Hydrogen Bonds per Molecular Weight

In order to compare the adsorption efficiency of the materials on the quartz surface, the number of hydrogen bonds formed under the unit molecular weight ($N_{unit}$) was introduced to characterize the number of hydrogen bonds per unit mass formed by the materials. The following formula was used to calculate:

(2) $$N_{unit}=\frac{N}{M}$$

where N is the number of hydrogen bonds formed; M is molecular weight.

The number of hydrogen bonds per unit molecular weight is shown in Table 3. It can be seen that under the same molecular weight conditions, the number of hydrogen bonds formed by PAA was the largest, and then decreased successively: PAM, PASP, PVAc, PU and HPMC. It shows that PAA can be better adsorbed on the surface of quartz, which is beneficial to improving the effect of sandy soil improvement.

This is similar to the results of Yan, L et al. 25 that the type of functional group is the main reason affecting the adsorption of polymers on substrates.

### 4.4. Mechanical Properties Analysis

**Figure 5:**
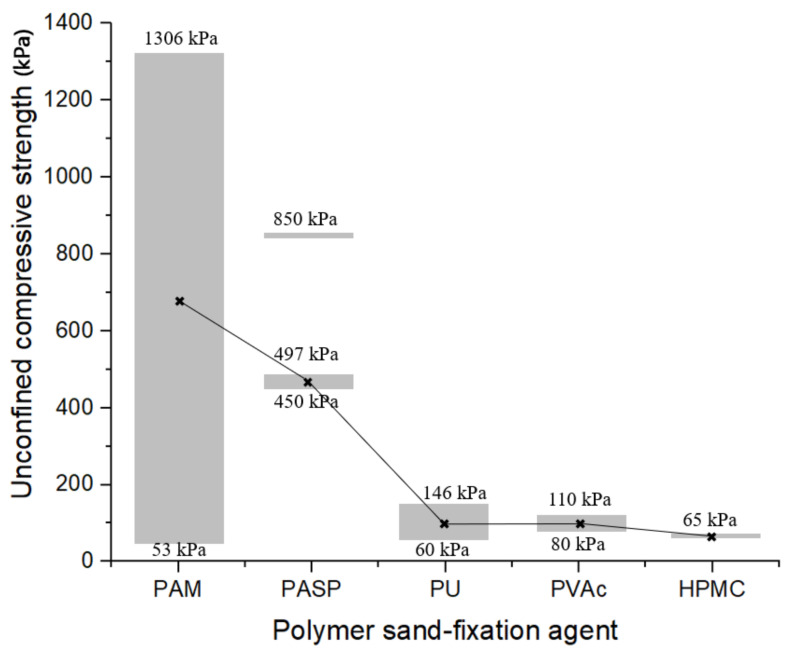
Unconfined compressive strength of sand-fixation agent (These marks are the midpoints of the values and connect them with lines.).

Polymers solidify sand by forming new physical and chemical bonds with the surface of sand particles [30]. The process is dominated by physical adsorption and does not involve chemical reactions. Therefore, the electrostatic attraction between hydrogen atoms and atoms with strong electronegativity (such as F, O) plays a leading role in the curing effect. Unconfined compression strength is a common index to measure the strength of sandy soil, which can effectively reflect the curing effect of sandy soil. The curing effect of the polymer mainly depends on its effective components. In this paper, the unconfined compressive strength of the sand-fixing agent with the above five materials (except PAA) as the main effective components was sorted out through literature collection (Figure 5). PAA is generally used as a water retaining agent, and its sand fixation principle is different from the other five polymers. The unconfined compressive strength of PAA modified sand has not been reported. When the mass fraction is 1%, the unconfined compressive strength under different curing time is: PAM (53–1306 kPa) [3] > PASP (450–497 kPa [31] or 850 kPa) [32] > PU (60–146 kPa) [19] ≈ PVAc (80–110 kPa) [33] > HPMC (65 kPa) [34]. The trend is positively correlated with the number of hydrogen bonds formed under the unit molecular weight, indicating that the number of hydrogen bonds can be used as one of the indicators to evaluate the improvement effect of sandy soil at molecular scale. It should be noted that the sand type will affect the strength of the improved soil, and the above conclusions can provide some basis for preliminary judgment. However, in practical engineering, the selection of materials requires strength test to calibrate the improvement effect.

## 5. Conclusions

The interaction between quartz and six common polymers, named PVAc, PU, HPMC, PASP, PAA and PAM, was studied by molecular dynamics simulation. The adsorption process of sand-fixing agent at molecular scale was studied by calculating the relative density distribution, hydrogen bond and binding energy of different atomic species in the polymer. The results show:

(1) The number of hydrogen bonds formed under the unit molecular weight is positively correlated with the compressive strength, and the number of hydrogen bonds can be used as one of the indicators to evaluate the improvement effect of sandy soil at molecular scale, which is helpful for the screening and development of new high-performance sand-fixing agents, which provides an accurate, efficient and inexpensive qualitative evaluation method for the curing effect of sand fixers and assists in the screening and development of new high-performance sand-fixers.
(2) Functional groups play an important role in the adsorption process. When the adsorption surface is quartz, the carboxyl group (PAA: −96.74 cal/mol) has the highest binding energy. The binding energy of hydroxyl group is the lowest (HPMC: −58.43 cal/mol). When the molecular weight was close, the binding energy of ester group (PVAc: −73.96 cal/mol) was slightly higher than that of peptide bond (PU: −70.56 cal/mol). Under the premise of chain structure, the binding energy of carboxyl group (PAA: −96.74 cal/mol) was higher than that of amide group (PAM: −74.01 cal/mol).
(3) This study was conducted under vacuum drying conditions, focusing on the effect of the material on sand particles after adsorption. However, the effect of water on adsorption cannot be ignored. In future research, more work will focus on the interaction between polymer molecules and quartz surface in aqueous solution.

## Figures and Tables

**Table 1:** Basic properties of sand-fixing agent.

Material Type	Molecular Weight (g/mol)	Chemical Formula	Functional Group	Structural Formula	References
PVAc	1292	C_60_H_92_O_30_	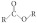 Ester group	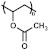	[18]
PU	1292	C_60_H_112_N_10_O_20_	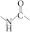 Peptide bond	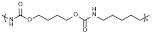	[19]
HPMC	1390	C_60_H_110_O_35_	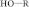 Hydroxyl	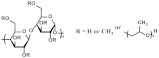	[20]
PASP	1734	C_60_H_70_N_16_O_45_	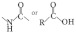 Peptide bonds and Carboxyl	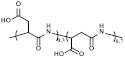	[21,22]
PAA	1442	C_60_H_82_O_40_	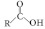 Carboxyl	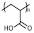	[4,23]
PAM	1422	C_60_H_102_N_20_O_20_	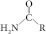 Amide group	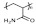	[3,24]

**Table 2:** Atomic types and their definitions in force field.

Atom Type	Description	Atom Type	Description
h1	Hydrogen, nonpolar	O2s	Oxygen, SP3, in esters
h1n	Hydrogen, bonded to N, Cl	O2h	Oxygen, SP3, in alcohol
h1o	Hydrogen, bonded to O, F	O2e	Oxygen, SP3, in ethers
o1=	Oxygen, SP2, in carbonyl	O2c	Oxygen, SP3, in acid
O2	Oxygen, SP3, generic	n3mh	Nitrogen, SP3, in amides with hydrogen

**Table 3:** The number of hydrogen bonds per unit molecular weight.

Type of Sand-Fixation Agent	PVAc	PU	HPMC	PASP	PAA	PAM
The number of hydrogen bonds (mol/g)	0.00387	0.00387	0.00216	0.0058	0.00902	0.00844

## Data Availability

The data and contributions presented in the study are included in the article. Further inquiries can be directed to the corresponding author.
